# Chemical and Nutritional Profiling of the Seaweed *Dictyota dichotoma* and Evaluation of Its Antioxidant, Antimicrobial and Hypoglycemic Potentials

**DOI:** 10.3390/md21050273

**Published:** 2023-04-27

**Authors:** Muhammad Imran, Arshad Iqbal, Syed Lal Badshah, Ayaz Ali Sher, Hammad Ullah, Muhammad Ayaz, Osama F. Mosa, Nada M. Mostafa, Maria Daglia

**Affiliations:** 1 Department of Botany, Islamia College University Peshawar, Peshawar 25120, Pakistan; imranbotany12345@gmail.com (M.I.); arshad.iqbal@icp.edu.pk (A.I.); ayazalisher@yahoo.com (A.A.S.); 2 Department of Chemistry, Islamia College University Peshawar, Peshawar 25120, Pakistan; shahbiochemist@gmail.com; 3 Department of Civil and Environmental Engineering, University of Toledo, Toledo, OH 43606, USA; 4 Department of Pharmacy, University of Napoli Federico II, Via D. Montesano 49, 80131 Naples, Italy; hammad.ullah@unina.it; 5 Department of Pharmacy, Faculty of Biological Sciences, University of Malakand, Dir(L), Chakdara 18000, Pakistan; 6 Public health Department Health Sciences College at Lieth, Umm Al Qura University, Makkah 21961, Saudi Arabia; 7 Biochemistry Department, Bukhara State Medical Institute Named after Abu Ali ibn Sino, Bukhara 20018, Uzbekistan; 8 Department of Pharmacognosy, Faculty of Pharmacy, Ain Shams University, Cairo 11566, Egypt; 9 International Research Center for Food Nutrition and Safety, Jiangsu University, Zhenjiang 212013, China

**Keywords:** seaweed, *Dictyota dichotoma*, GC-MS, FTIR, antimicrobial, antioxidant, hypoglycemic agent

## Abstract

Seaweed has been known to possess beneficial effects forhuman health due to the presence of functional bioactive components. The *n*-butanol and ethyl acetate extracts of *Dictyota dichotoma* showed ash (31.78%), crude fat (18.93%), crude protein (14.5%), and carbohydrate (12.35%) contents. About 19 compounds were identified in the *n*-butanol extract, primarily undecane, cetylic acid, hexadecenoic acid, *Z*-11-, lageracetal, dodecane, and tridecane, whereas 25 compounds were identified in the ethyl acetate extract, mainly tetradecanoic, hexadecenoic acid, *Z*-11-, undecane, and myristic acid. FT-IR spectroscopy confirmed the presence of carboxylic acid, phenols, aromatics, ethers, amides, sulfonates, and ketones. Moreover, total phenolic contents (TPC) and total flavonoid contents (TFC) in ethyl acetate extract were 2.56 and 2.51 mg GAE/g and in *n*-butanol extract were 2.11 and 2.25 mg QE/g, respectively. Ethyl acetate and *n*-butanol extracts at a high concentration of 100 mg mL^−1^ showed 66.64 and 56.56 % inhibition of DPPH, respectively. Antimicrobial activity revealed that *Candida albicans* was the most susceptible microorganism, followed by *Bacillus subtilis, Staphylococcus aureus*, and *Escherichia coli*, whereas *Pseudomonas aeruginosa* showed the least inhibition at all concentrations. The in vivo hypoglycemic study revealed that both extracts exhibited concentration-dependent hypoglycemic activities. In conclusion, this macroalgae exhibited antioxidant, antimicrobial, and hypoglycemic potentials.

## 1. Introduction

Marine algae are one of the largest producers of biomass in the marine environment [1]. They generate a wide range of chemically active compounds in their environmentwhich exhibit antibacterial, antifungal, antimacrofouling, and other therapeutic properties [2,3]. Seaweeds are commonly consumed as food in Asian countries even though they are now the basis for numerous industrial products including agar, algin, and carrageenan [4]. Oceans around the world include seaweeds in a stunning array of attractive shapes, colors, and sizes [5]. They are typically prevalent in rocky, shallow coastal locations, particularly those that are exposed during low tide. Sincethe beginning of time, coastal populations around the world have harvested and consumed sea vegetables [6]. Diverse aquatic species find them to be a perfect home and asource of food, shelter, and vegetation. Based on the type of pigments and morphological, anatomical, and reproductive features, seaweeds are divided into different groups including Phylum Chlorophyta (Class Ulvophyceae), Phylum Rhodophyta, and Phylum Ochrophyta (Class Phaeophyceae) [7].

*Dictyota* is a genus of the family Dictyotaceae, which is known to bear a cosmopolitan nature. Dictyotales species (brown algae) produce a variety of bioactive secondary metabolites with broad antiherbivore effects in marine environments. *Dictyota* species are rich in phytoconstituents, mainly of the terpene class. Many compounds (about a third) identified from brown algae were reported from different *Dictyota* species [8]. The most prevalent member of this family is one of the major seaweeds, *Dictyota dichotoma*. It has been extensively studied, though the studies have identified a wide variety of differences among its contents depending on the time and location of the collection. From the Dictyotaceae family, this species is responsible for the highest proportion of versatile bioactives, particularly diterpenes. Several bioactives from *D. dichotoma* have been previously reported, including two compounds, dictyohydroperoxide and hydroperoxyacetoxycrenulide, containing hydroperoxyl groups rarely found in algal terpenoids, and two diterpenoids, namely pachydictyol B and pachydictyol C [9]. Many researchers have recently given a great deal of attention to the genus, due to its economic significance as an animal feed and antibiofouling and medicinal agent, overthe past 10 years [10]. However, research exploring seaweed resources in Pakistan is poorly elicited although it has enormous potential [11]. The majority of research has been on proximate and biochemical analyses of seaweed [12]. There are very few studies in the literature on seaweeds collected from the Pakistani coast that analyze the phytochemical composition and antimicrobial and antioxidant properties [13]. Season, age, species, and geographic location all affect the yield and composition. 

Subsequently, in the current phytochemical study, *n*-butanol and ethyl acetate extracts of *D. dichotoma* were subjected to GC-MS analysis for the identification of volatile metabolites, and an FT-IR spectroscopic approach to identify the presence of functional groups of different classes of compounds for the first time from the Pakistani chemotype. The total phenolics (TPC) and total flavonoid content (TFC) are presented herein for both extracts and their in vitro antioxidant and antimicrobial activities were tested. Moreover, the hypoglycemic potential of the algal extracts was evaluated for the first time.

## 2. Results

### 2.1. Proximate Nutrient Composition

**Figure 1:**
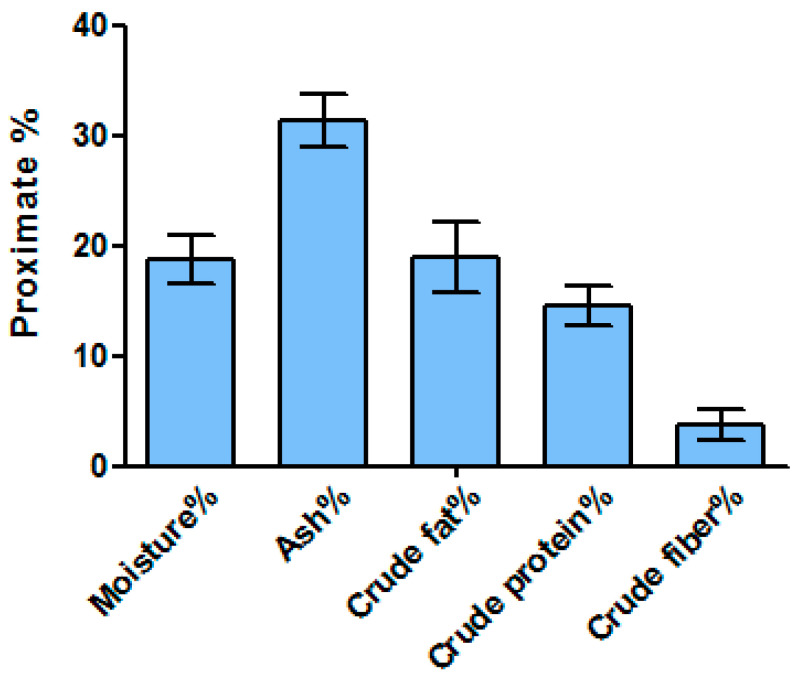
Proximate content analysis of *D. dichotoma*. Measurements were performed in triplicate.

Considerable differences were observed during the proximate nutrient contents among species (Figure 1). Ash was recorded as 31.78% followed by crude fat and crude protein at 18.93% and 14.5%, respectively. Carbohydrate content was calculated at 12.35% by a subtraction method ona wet basis. The crude fiber was recorded as at least 3.66%, whereas the sample contained 18.78% moisture. The energy content was also determined by calculative value showing a high energy value of 137.06 Kcal/100 g.

### 2.2. GC-MS Analysis 

About 25 compounds were tentatively detected in the ethyl acetate of *D. dichotoma* through GC-MS analysis (Table 1, Figure S1). The most abundant compound was detected as tetradecanoic acid (C_14_H_28_O_2_), constituting 33.78% of the total extract. The second most abundant compound was noted as hexadecenoic acid, *Z*-11- (10.98%), followed by undecane, myristic acid, tridecane, 4,8-dimethyl-, and isoaromadendrene epoxide.

Similarly, about 19 compounds were tentatively detected in the *n*-butanol extract through GC-MS (Table 2, Figure S2). The major compounds were identified as undecane (14.98%), cetylic acid (14.62%), hexadecenoic acid, *Z*-11- (9.24%), lageracetal (8.62%), dodecane (8.57%), and tridecane (6.57%). 

### 2.3. FTIR Spectrum Analysis

**Figure 2:**
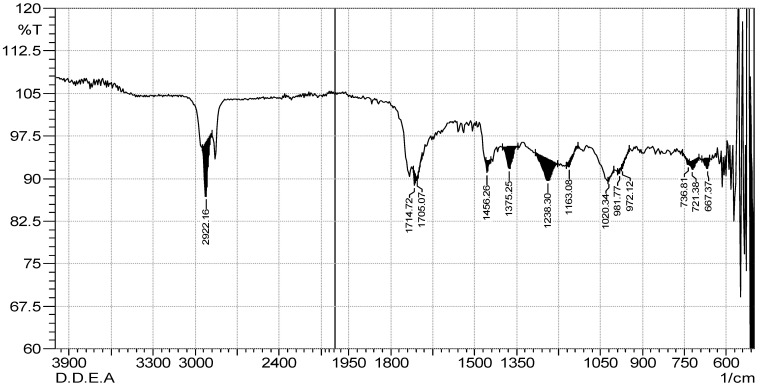
FTIR spectrum of ethyl acetate extract of *D. dichotoma*.

**Figure 3:**
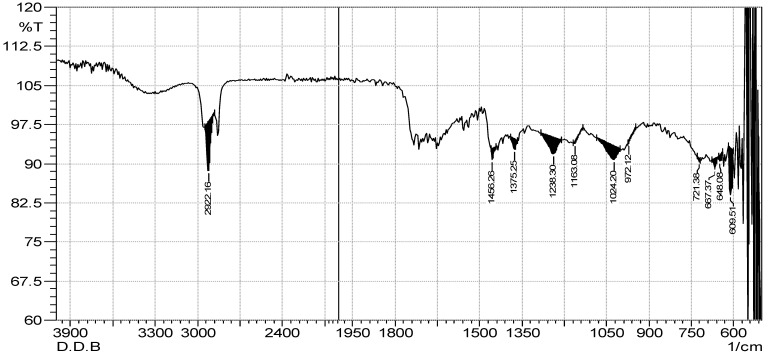
FTIR spectrum of *n*-butanol extract of *D. dichotoma*.

The results of the FTIR spectrum of both extracts confirm different types of phytochemical constituents. Table 3, Figure 2 and Figure 3 demonstrate 12 functional groups which were recognized from the ethyl acetate and *n*-butanol extracts. An intense peak at 2922.16 cm^−1^ was recorded for both extracts, which was assigned to the asymmetric stretching of -CH(CH_2_) vibration, indicating that the extract contained saturated aliphatic compounds such as lipids, followed by a low band ranging from 1714.72 to 1705.07 cm^−1^, confirming the C=O functional group of carbonyl compounds in both extracts. Similarly, aromatic compounds werealso detected in the extract by recording the peak of 1456.26 cm^−1^, which is assigned to the C=C. Certain types of other groups, i.e., phenol or tertiary alcohol, acids and amines, and alkyl halides, were absorbed in 1375.25 cm^−1^, 1238.30 cm^−1^, and 1163.08 cm^−1^ in both extracts. However, in the ethyl acetate and *n*-butanol extracts, phosphate ions were absorbed at 1020.34 cm^−1^ and 1024.20 cm^−1^, and alkene at 972.22 cm^−1^ and 736.81 cm^−1^, respectively. Halogen compounds (chloro-compound and iodo-compound) were absorbed at 721.38 cm^−1^ and 667.37 cm^−1^ in both extracts, respectively.

### 2.4. Estimation of TPC and TFC

The regression equation of the calibration curve was used to compute the TPC and TFC of the extracts, which were then represented as mg of gallic acid equivalents (GAE) and mg of quercetin equivalents (QE) per gram of sample in dry weight (mg/g). TPC values in the extracts were essentially the same. The extract of ethyl acetate, 2.56 ± 0.34 mg GAE/g, had the highest TPC value, followed by the extract of *n*-butanol, 2.51 ± 0.67 mg GAE/g (Table 4). Similarly, *n*-butanol extract had a TFC of 2.25 ± 0.28 mg QE/g, whereas ethyl acetate had 2.11 ± 0.89 mg QE/g.

### 2.5. DPPH Anti-Radicals Assay

**Figure 4:**
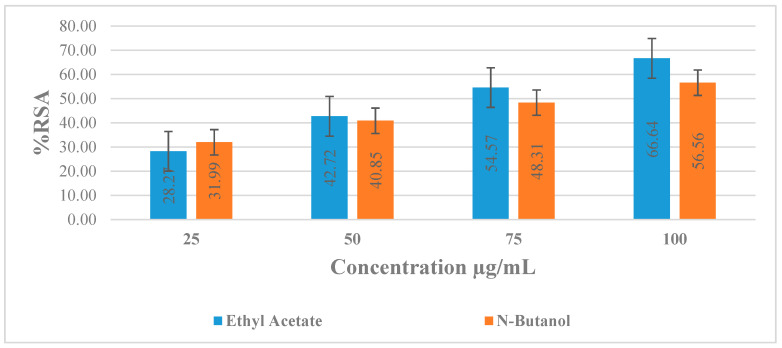
Percent radical scavenging effects of various extracts from *D. dichotoma*.

In the current investigation, the DPPH assay was used to assess the antioxidant potential of two extracts of *D. dichotoma*. Table 4 and Figure 4 show the results for DPPH activity. Generally, the percentage of RSA activity was higherin the ethyl acetate extract than in the *n*-butanol of *D. dichotoma*. In the ethyl acetate extract, % RSA ranged from 28.17% to 66.64%. However, in butanol extract, % radical scavenging activity ranged from 31.99% to 56.56%. The highest concentration 100 µg mL^−1^ showed a higher % RSA, followed by lower concentrations 75, 50, and 25 µg mL^−1^, i.e., 66.64%, 54.57%, 42.72%, and 28.27%, for ethyl acetate in the tested species. Similarly, 31.99%, 40.85%, 48.31%, and 56.56% RSA were recorded for 25, 50, 75, and 100 µg mL^−1^ concentrations of *n*-butanol extract. IC_50_ values were calculated for each concentration, which indicates that ethyl acetate showed good activity as compared to butanol because *n*-butanol’s IC_50_ was higher than ethyl acetate.

### 2.6. Antimicrobial Activity

**Figure 5:**
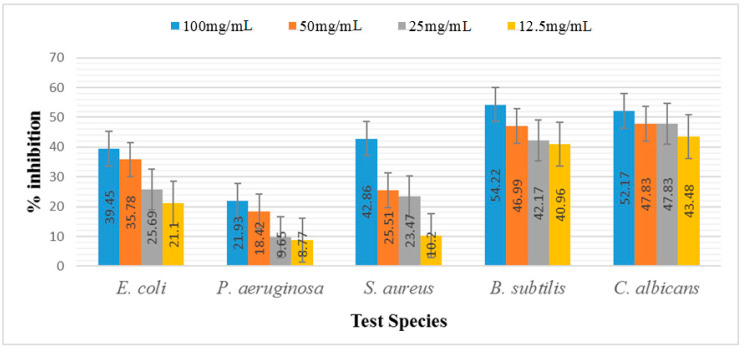
Antimicrobial potentials of ethyl acetate fraction of *D. dichotoma*.

**Figure 6:**
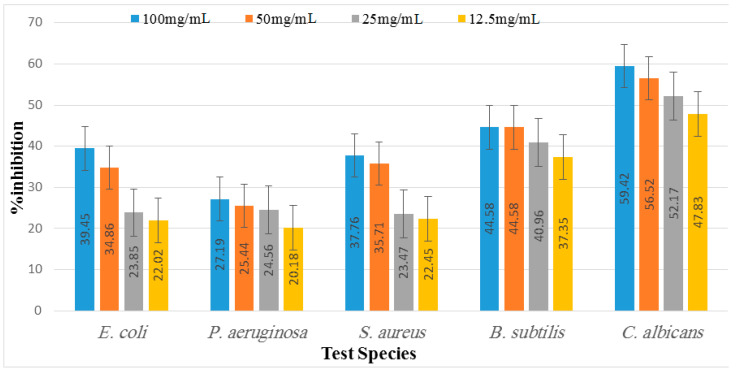
Antimicrobial activity of *n*-butanol extract of *D. dichotoma*.

The antimicrobial potential of *D. dichotoma* ethyl acetate and *n*-butanol extracts against various bacterial and fungal strains are shown in Table S1 and Figure 5 and Figure 6. It was observed that the least potent tested extract was *n*-butanol, followed by the ethyl acetate extract. Moreover, among the bacterial strains, *B. subtilis* was recorded as the most susceptible organism, followed by *S. aureus* and *E. coli*, whereas *P. aeruginosa* showed the least inhibition at all concentrations. Maximum inhibition was recorded at the concentration of 100 mg mL^−1^, followed by 50 mg mL^−1^, 25 mg mL^−1^, and 12.5 mg/mL, respectively. In the case of *B. subtilis*, 54.22%, 46.99%, 42.17%, and 40.90% inhibition were recorded for 100 mg mL^−1^, 50 mg mL^−1^, 25 mg mL^−1^, and 12.5 mg mL^−1^ of ethyl acetate extract, respectively. Butanol extract, on the other hand, trailed behind when measuring, and showed 44.58%, 44.58%, 40.96%, and 37.35% inhibition at 100, 50, 25, and 12.5 mg mL^−1^, respectively, for the same bacterial strain. In the case of *S. aureus,* the same manner of inhibition was recorded. Similarly, maximum inhibition was recorded for 100 mg mL^−1^ of ethyl acetate, i.e., 42.86%, whereas 50 mg mL^−1^ and 12.5 mg mL^−1^ of *n*-butanol showed more inhibition, i.e., 35.71% and 22.45% compared to the ethyl acetate extract (25.51% and 10.2%), respectively. For ZOI in *E. coli,* 39.45% was recorded for the 100 mg mL^−1^ concentration of both ethyl acetate and *n*-butanol extract, followed by 50 mg/mL, which showed 34.86% and 35.78% ZOI, respectively. The least ZOI in *E. coli* was recorded for lower concentrations (25 mg mL^−1^), i.e., 25.69% and 23.85%, and 12.5 mg/mL, i.e., 21.01% and 22.02% of both ethyl acetate and *n*-butanol extracts, respectively. The present study reports comparatively enhanced activities for the ethyl acetate extract, which relates to the stronger activity of the ethyl acetate extract than the *n*-butanol. *P. aeruginosa*, on the other hand, was the least resistant specie against both extracts, and showed 27.19%, 25.44%, 24.56%, and 20.18% ZOI at 100, 50, 25, and 12.5 mg mL^−1^, respectively. Similarly, ethyl acetate showed 21.93%, 18.42%, 9.65%, and 8.77% ZOI at the same concentrations discussed above, respectively. A comparison of the results of *C. albicans* showed a non-significant variation between zones of inhibition at α< 0.001, 0.01. Among the extracts, ethyl acetate had the least inhibition, whereas *n*-butanol had significantly higher zones of inhibition at α < 0.05. We found a positive correlation between the increasing concentrations and zones of inhibition for *C. albicans*. The 100 mg mL^−1^ concentration had the highest ZOI, i.e., 59.42% and 52.17% by *n*-butanol and ethyl acetate, respectively, for *C. albicans* and is hence the most effective concentration, followed by 50 and 25 mg mL^−1^, whereas the least ZOI was recorded for lower concentrations (12.5 mg mL^−1^), i.e., 47.83% and 48.43%, by *n*-butanol and ethyl acetate, respectively. There was a positive and significant relation between the increasing concentrations and the zone of inhibition of *C. albicans*.

### 2.7. Hypoglycemic Activity

Diabetes is a metabolic condition in which blood glucose levels consistently rise, which is commonly called hyperglycemia. Long-term hyperglycemia can cause neuropathy, nephropathy, amputations, and other complications if the appropriate therapy is not received [14]. *D. dichotoma* extracts were screened for antidiabetic activity and compared to a control diabetic group in the current study (Table 5). The results of the study revealed that the animals of the diabetic control group showed significantly raised blood glucose levels (340.50 ± 7.99) on the thirdday after the injection of alloxan to Group 2 when compared with normal animals (99.17 ± 6.68). A substantial decline in glucose level was noted when the diabetic animals were cured with the standard drug glibenclamide and extracts (ethyl acetate and *n*-butanol) of *D.dichotoma* when compared with the control as given in Table 5.

The lowering of blood glucose byethyl acetate extracts at 100 mg kg^−1^ was observed at 6 h 300 mg kg^−1^ 291.00 ± 13.54* followed by 200 mg kg^−1^ and 100 mg kg^−1^ (271.33 ± 8.66* and 305.00 ± 7.59*), respectively. After 24 h, a significant lowering at α > 0.05 of blood glucose was observed, i.e., 235.67 ± 7.00* for 300 kg, 266.50 ± 7.71* for 200 mg kg^−1^, and 294.00 ± 17.78* for 100 mg kg^−1^. The lowering of blood glucose by the *n*-butanol extract of *D. dichotoma* at 6 h was 229.17 ± 5.34 for 300 mg kg^−1^ followed by 200 and 100 mg kg^−1^ (239.67 ± 5.13* and 248.67 ± 5.68*). Similarly, the extract showed a significant decrease at α > 0.05 in the blood glucose level, and at 24 h it was 216.67 ± 9.14* for 300 mg kg^−1^, followed by 233.67 ± 6.80* and 245.83 ± 4.75* for 200 and 100 mg kg^−1^. All extracts were as useful as a standard drug that is available in the market, i.e., glibenclamide. The onset of the anti-diabetic action of glibenclamide was at 6 h (224.67 ± 8.69) and at 24 h it reached 107.83 ± 7.78.

## 3. Discussion

Diverse kinds of seaweeds havelong been used traditionally in the diets of Asians. The nutritional profiles of numerous seaweeds have shown that they are excellent sources of fatty acids, protein, dietary fiber, and several minerals [15]. There are many different types of seaweeds used, not just for human consumption but also as a component of animal and poultry feed [16]. Recently, the demand for some edible seaweed has surged in North America, South America, and Europe due to its greater nutritious components [17]. The usage of herbal medicines for medical treatment has grown, and it is now crucial to screen medicinal plants for bioactive components [18]. Secondary metabolites are abundant in herbal products [19]. GC-MS is typically used to gather information and examine medicinal plants to identify their bioactive components. The GC-MS of *D. dichotoma n*-butanol and ethyl acetate extracts revealed the presence of a variety of therapeutically useful chemicals. Bioactive molecules such as undecane, 3,7,11,15-tetramethyl-2- hexadecen-1-ol, pentadecanoic acid, 14-methyl-, methyl, octadecenoic acid, methyl ester, (Z)-, palmitic acid, methyl ester, phytol, etc. have various biological activities. Undecane is used as an enzyme inhibitor and antimicrobial, while 3,7,11,15-tetramethyl-2- hexadecen-1-ol has anti-inflammatory, antipyretic and antinociceptive potential, whereas pentadecanoic acid, 14-methyl-, methyl ester has antimicrobial and antioxidant properties. Similarly, 6-octadecenoic acid, methyl ester, (Z)- is used as an antioxidant and antimicrobial. Palmitic acid, methyl ester has antitumor potential while phytol has antimicrobial, anti-inflammatory, anticancer, and diuretic properties, and is used for resistant gonorrhea, joint dislocation, headache, hernia, and as a stimulant [20].

Similarly, according to the proximate analysis, seaweed contains considerable amounts of key nutrient proteins, fat, carbohydrates, and fibers. The current results are consistent with an earlier study published in [21], which found nearly the same results regarding the nutrition of *D. dichotoma*. Seaweed might be a great source of dietary protein, even though we did not profile the key amino acids. According to [22], seaweeds are known to have a reduced protein content, which is consistent with the findings of the present study. Similarly, it is also reported that *D. dichotoma* contains 7.28 ± 0.25% proteins and 25.35 ± 0.32% carbohydrate, which is in agreement with the present finding [22]. The low crude fiber content observed in our study (3.66%) was in agreement to those reported for other brown algae such as *Padina minor* (3.81%), *Sargassum oligocystum* (6.49%)*,* and *Sargassum polycystum* (6.52%) [23,24]. According to Mwalugha et al. [24], the factors affecting variations in crude fiber content in seaweeds can include photosynthetic activity differences among species, growth stage variations, and season of collection, which affect their nutritional uptake from surrounding ecosystem. In the present study, the moisture content, ash, and crude fats were also calculated, which were 18.78%, 31.78%, and 18.93%, respectively. These results were in accordance with Gokulakrishnan et al., who reported comparable results for the moisture content (4.23 mg g^−1^), ash (9.47 mg g^−1^), and crude fats (19.23 mg g^−1^) [25].

It is worth noting that the mineral composition of *D. dichotoma* was previously reported by Deyab et al. (2017), stating that macroelements analysis showed high contents of potassium, sodium, calcium, and phosphorous, respectively [26]. Magnesium was the least-detected macroelement. According to their study, Mn and Fe were the major microelements detected in *D. dichotoma*.

The objective reflection of componential disparities is reflected in spectral differences. We can determine the origin of various extracts precisely and effectively utilizing the macroscope fingerprint properties of the FT-IR spectrum, trace the elements in the extracts, identify the medicinal materials, and even assess the qualities of medicinal materials [27]. Therefore, the FT-IR spectrum is the most reliable way to assess and identify chemical constituents in complex systems [28]. The FT-IR analysis revealed the characteristic infrared absorbance. The listed infrared functional group absorptions characteristic was cited from the literature. Both the crude extracts of *D. dichotoma* exhibited similar functional groups, such as saturated aliphatic comp., lipids, carbonyl, aromatic substances, phenol or tertiary alcohol, acid, amine, amine, alkyl halides, phosphate ion, andalkene and halogen compounds.

The presence of oxygen-containing aromatic compounds has been reported to be associated with potential antioxidative capabilities [29]. Additionally, compounds possessing phenolic groups are reported to exhibit promising biological activities, such as antioxidant, antimicrobial, anti-inflammatory, and antidiabetic activities [30,31]. The presence of lipids in *D. dichotoma* as indicated from the FT-IR spectrum may be correlated to the observed antimicrobial activity; according to Fischer et al. [32] certain fatty acids can even be selective. Many algae are reported to produce halogen-containing compounds, this isin accordance with the chloro- and iodo-functional groups detected in *D. dichotoma* extracts. Halogenated compounds have shownantimicrobial activities in the previous literature [33].

Secondary metabolites such as phenolic and flavonoid molecules are indirectly involved in physiological processes [34]. Many studies have focused on phenolic and flavonoid isolation, characterization, and pharmacological potentials [35]. Mostly, flavonoids and phenolics are proven to have positive pharmacological properties [36]. The flavonoids and phenolic content and antioxidant activity in *D. dichotoma* are presented (Table 2). The TPC in the ethyl acetate extract was higher compared to the *n*-butanol of *D. dichotoma*. This fact is due to the type of solvent that selectively affects the phenolic compounds [37]. This is in agreement with [38], who reported that the TPC value of extracts of *D. dichotoma* was found to be 2.02 ± 0.11 mg GAE g^−1^, and that of methanol was 2.14 ± 0.15 mg GAE g^−1^. Similarly, in another study, the TPC value of ethanol extract of *D. dichotoma* was 69.5 ± 0.7 mg of GAE g^−1^ [34], and 0.851 ± 0.06 mg of GAE g^−1^ of TPC was reported by [39]. Similarly, more studies also revealed the TPC in the different extract of *D. dichotoma*, such as [40], who reported the phenolic contents of six Dictyotales. On the other hand, researchers reported the biochemical constituents of seaweeds, in which they recorded the TFC value of *D. dichotoma* as 3.42 ± 0.1, which is almost in agreement with the present results [41]. Similarly, the results were also following the results of previous studies where the TFC contents of *Dictyota* species were reported [22,42].

A test compound’s ability to neutralize free radicals produced independently by any enzymatic or transitional metal-based mechanism is demonstrated by the bleaching of DPPH solution. A persistent free radical called DPPH is reacted with antioxidants to produce 1,1-diphenyl-2-(2,4,6-trinitrophenyl) hydrazine. The degree of decolorization reveals how well the antioxidant molecule scavenges free radicals [43]. In the present study, ethyl acetate was found to show significantly stronger antioxidant activity and contained a good amount of TPC and TFC as confirmed by a test performed with a photospectrometer and FTIR spectroscopy through functional groups. Measured levels of % RSA activity in extracts of *D. dichotoma* were comparable with previous studies [44,45]. The extract of *D. dichotoma* was shown to display antioxidant activity as evaluated by the DPPH radical scavenging activity and was attributed to its phenol, diterpenoid, phlorotannin, vitamin E, carotenoid, and vitamin C contents [46,47]. Diterpenoids within *D. dichotoma* were reported to have potent antioxidant effects as evaluated by the ABTS and erythrocytes hemolysis activities [48]; however, fucoxanthin was an active radical scavenger, displaying 13.5 times higher hydroxyl radical scavenging activity compared to that of vitamin E [49]. The use of the DPPH test alone in our investigation restricts the capacity to suggest a view point on the mechanism of the observed low antioxidant activity of the extract due to the documented lack of correlation between the numerous methods used to evaluate the antioxidant capacities of extracts.

The ability of the seaweed to produce bioactive secondary metabolites was hypothesized to be indicated by the synthesis of antibacterial active chemicals [49]. In this study, algal extracts were produced and tested for their antibacterial properties against microbes. The measured microbial growth was inhibited to varying degrees by all different types of algal extracts. The findings showed that the examined bacteria were only moderately resistant to the algal extracts. Among the bacterial strains, *B. subtilis* was recorded as the most susceptible organism, followed by *S. aureus* and *E. coli*, whereas *P. aeruginosa* was found to be more resistant. It was claimed that compared to Gram-negative bacterial strains, Gram-positive bacterial strains were more vulnerable to seaweed extracts [49]. Another study reported that the antimicrobial compound present in the marine seaweed (*Dictyota acutiloba*) has a more potent antagonistic effect towards Gram-positive bacterial pathogens [50]. In addition, they showed a powerful antimicrobial effect on *B. subtilis* bacteria. Moreover, a similar order of activity for the extracts of brown algae has been reported by [51], although they used different crude extracts to thoseemployed in this study. Polyhydroxylated fucophlorethol, an antibacterial chemical derived from the brown alga *Fucus vesiculosus*, was found to be effective against both Gram-positive and Gram-negative bacteria. A particular bacterial group’s vulnerability resulted from differences in the makeup and structure of their cell walls [52]. However, medicines and other environmental contaminants are blocked by Gram-negative bacteria’s outer membrane. This might be as a result of antibiotics acting as competitive inhibitors of the transpeptidase required by the bacteria to constructa cell wall after penetrating the outer membrane of (primarily Gram-negative) bacteria via porins, which ultimately results in cell lysis and decreases the pathogens’ capacity to successfully replicate. Contrarily, *Dictyota dichotoma* extracts are believed to have antimicrobial actions that may be brought on by altering the cell membranes of the pathogens that are being targeted, with changes to the cell envelope leading to defective control of osmolality and ultimately cell death. Despite the fact that this is a preliminary study, detailed investigations to discover the compositions of each extract are required to find the main components of marine algae that may operate as potent antimicrobials [53]. Compared to water-based techniques, organic solvents offer a higher efficiency in the extraction of chemicals for antibacterial activity [54]. This statement was justified by researchers who demonstrated comparable activities of *Dictyota barteyresiana* extract against bacterial strains [55]. The relatively higher activities of the organic solvent extracted samples conform with previous reports [56]. Moreover, the antifungal results of *C. albicans* showed a non-significant variation between zones of inhibition. Among the extracts, ethyl acetate had the least inhibition, whereas *n*-butanol had significantly higher zones of inhibition in antifungal activity. This significant effect of *n*-butanol extract might be due to the presence of some pharmacologically active compounds present in the extract, such as 6-Octadecenoic acidmethyl ester (Z)-, which is used as an antioxidant and antimicrobial. Palmitic acid methyl ester has antifungal, antibacterial, and antitumor potential, while phytol has strong antimicrobial, anti-inflammatory, anticancer, and diuretic properties [20]. Numerous studies are being conducted to evaluate different compounds through GC-MS and the antioxidant and antimicrobial potential and chemical compositions of *Peperomia pellucid* leaf extract. The results of the study revealed that phytol (37.88%) was the major compound in the plant extract, followed by hexadecanoic acid methyl ester (18.31%) and 9,12-octadecadienoic acid (Z, Z)- methyl ester (17.61%). The findings from this study indicated that the methanol extract of *P. pellucida* leaf possesses vast potential as a medicinal drug [55]. Similarly, another study evaluated the GC-MS analysis of *Bunchosia armeniaca* and revealed the presence of phytochemicals such as 9,12,15-octadecanoic acid, methyl ester (Z,Z,Z) and *n*-hexadecanoic acid, which showed the highest antioxidant potentials [57]. The present study showed that the extract had high antimicrobial as well as antioxidant potential. This statement may be linked with the present investigation of GC-MS for both extracts, for which we also reported the same compounds as those mentioned above, which may be responsible for such activities.

Hyperglycemia is a feature of the metabolic illnesses known as diabetes mellitus. These metabolic illnesses involve modifications to the metabolisms of carbohydrates, fats, and proteins linked to absolute or relative insulin secretion and/or action deficits. Diabetes is characterized by polyuria, polydipsia, polyphagia, pruritus, and sudden weight loss, among other symptoms. Due to the negative side effects of using insulin and oral hypoglycemic medications, patients are increasingly requesting to use natural items with antidiabetic activity (OHAs) [58]. In this study, diabetic rats’ blood glucose levels significantly decreased after exposure to *Dictyota dichotoma* ethyl acetate and *n*-butanol extract at doses of 100, 200, and 300 mg kg^−1^, but normal rats were unaffected. After 24 h of drug treatment, the extract had a greater blood-glucose-lowering impact on diabetic rats than the oral hypoglycemic medication glibenclamide. This alga contains a variety of substances, including phenols, alkaloids, and other substances, according to phytochemical screening. In the current investigation, we found that diabetic rats receiving 300 mg kg^−1^ of both *Dictyota dichotoma* extracts had the greatest decreases in blood glucose. This result is in agreement with previous researchers who also reported the hypoglycemic effects of bioactive compounds from marine macroalgae [59]. Similarly, another study reported that marine organisms produce a large array of natural products with relevance in drug discovery [60]. Natural products including phenolics are reported to show promising biological activities, including antioxidant, antibacterial, antitumor, antivirus, anticoagulant, anti-inflammatory, hypotensive, antidiabetic, and others [61,62,63]. In the current investigation, GC-MS analysis identified several distinct phytochemicals, including 19 compounds in *n*-butanol extract and 25 compounds in ethyl acetate. Another studies reported that tetradecanoic acid, phytol acetate, trans phytol, *n*-hexadecanoic acid, and 9 Z,12 Z-octadecadienoic acid were among the several bioactive chemicals found in the leaf essential oils and fruit ethanolic extracts of *Ficus carica* upon a gas chromatography-mass spectroscopy study [64,65]; the findings of this investigation imply that the ethanolic extract of *F. carica* fruit may have antidiabetic potential [64]. Similarly, researchers reported the chemical profiling by GC–MS analysis of *Leucophyllum frutescens*, which revealed that majorly it contains 9-octadecenoic acid (Z)-, methyl ester, pentadecanoic acid, 14-methyl-, methyl ester, 9,12-octadecadienoic acid, methyl ester, 9,12,15-octadecatrienoic acid, *n*-hexadecanoic acid, hexadecanoic acid, ethyl ester, and phytol which showed significant antioxidant, antidiabetic, and cytotoxic activities [66].

## 4. Materials and Methods

### 4.1. Collection and Extraction

The macroalga *D. dichotoma* was collected in December (winter season) from the French bay beach (coastal areas) of Karachi. To remove the sand particles, the gathered materials were rinsed with tap water. They were left in the shade to dry at a temperature between 25 and 35 °C. The dried material was then crushed with an electrical grinder into fine powder. About 300 gm of the selected macroalgae was dissolved in 1000 mL of solvents and was kept for 21 days and the filtrate was obtained. The solvents *n*-butanol and ethyl acetate were used for extraction [67].

### 4.2. Proximate Nutrient Composition

The AOAC procedures were used to determine the ash, crude fiber, crude fat, moisture, and crude protein content of algal samples [68]. The percentages of moisture, crude fat, ash, crude fiber, and crude protein were added together, and the percentage of carbohydrates was calculated by subtraction from a total of one hundred. Using the “Atwater factor”, the amount of carbohydrate, fat, and crude protein was multiplied by 3, 9, and 3, respectively, to obtainthe amount of energy. The product was then added [69].

### 4.3. Gas Chromatography-Mass Spectrometry (GC-MS) Analysis

Gas chromatography-mass spectrometry (GC-MS) analysis was carried out (model; Japan, Kyoto, Shimadzu Corporation, QP2010 Ultra) on a capillary column with a 0.25 mm inner diameter and a 30 m length. The stationary phase used was of 0.25 mm film thickness (U.S.A, Restek Corporation, Rtx-5MS, Bellefonte, PA, USA). Helium (99.999%) was employed as the carrier gas, moving at a constant speed of 36.3 cm/s. A sample volume of 1 l was injected using the AOC-20i + s auto-injector. At 290 °C, the injection port was maintained in split-less mode. The GC oven was preheated to the following temperature: 5 min at 50 °C, followed by 10 min of holding at 300 °C at a rate of 2 °C/min. The *m*/*z* range of 30 to 700 was used to construct a total ion chromatogram. By comparing their mass spectra to the National Institute of Standards and Technology’s database (NIST), and with the literature, GC peaks were identified [70,71]. By comparing each constituent’s peak area to the chromatogram’s overall peak area, the relative percentage quantity of each constituent was calculated [72].

### 4.4. FourierTransform Infrared Spectroscopy (FTIR)

Perkin-Elmer Fourier transform infrared (FTIR) spectrophotometer was used for the FTIR analysis. The KBr salts were combined with the tested extracts using a mortar, and thin pellets were created by compression. Each sample was independently placed into the FTIR spectroscope (PerkinElmer FTIR2000, Waltham, MA, USA). The average of two separate observations from 4000 to 400 cm^−1^ with 128 scans, each at a resolution of 2 cm^−1^, was used to create each spectrum.

### 4.5. Total Phenolic and Flavonoid Content

#### 4.5.1. Solution Preparation

A 1% solution of gallic acid (10 mg mL^−1^), often known as standard 1 solution, was producedby dissolving 1 g of gallic acid in 100 mL of methanol. To create a 1% solution of quercetin (10 mg mL^−1^), often known as standard 2 solutions, 1 g of quercetin was individually dissolved in 100 mL of methanol [73].

#### 4.5.2. Total Phenolic Content (TPC)

The total phenolic content of both extracts was determined using the Folin–Ciocalteu method [74]. A standard gallic acid curve was produced using the dilutions of (0.1, 0.5, 2.5, 1.0, and 5 mg mL^−1^) in methanol from the standard 1 solution of gallic acid. Each of these solutions, 100 µL, was added to 500 µL of water, followed by 100 µL of Folin–Ciocalteu reagent, and allowed to stand for 6 min. The reaction mixture was then given a final addition of 1 mL of sodium carbonate at 7%. After 90 min, the absorbance at 760 nm was spectroscopically measured. The amount of gallic acid equivalents (mg GAE g^−1^) was used to measure the total phenolic content. There were three duplicates of each experiment [73].

#### 4.5.3. Total Flavonoids Content (TFC)

The total flavonoid content of the extracts was assessed using an assay for the formation of an aluminum chloride complex. Flavonoid content’s quercetin equivalent was calculated using quercetin as the standard. This required the creation of a calibration curve for quercetin. In methanol, dilutions of (0.1, 0.5, 1.0, 2.5, and 5 mg mL^−1^) concentrations of the standard quercetin solution 2 were created. After mixing 100 µL of each quercetin dilution with 500 µL of distilled water and 100 µL of 5% sodium nitrate, the mixture was left to stand for 6 min, then 150 µL of 10% aluminum chloride solution was mixed and allowed to stand for 5 min, and then 200 µL of a 1M sodium hydroxide solution was successively added. On a UV spectrophotometer, the reaction mixture’s absorbance was measured at 510 nm. The process was carried out again using the extracts, and the total flavonoid concentration was determined as mg QE g^−1^ of quercetin equivalents. There were three duplicates of each technique [73].

### 4.6. Antioxidant Activity

#### DPPH Anti-Radicals Assay

*D. dichotoma* extracts were tested for their capacity to scavenge DPPH radicals [75,76]. The stable radical DPPH in ethanol exhibits a deep violet hue. When it reacts with a hydrogen donor, its color is bleached. An amount of 0.1 mL of each extract was added to 2 mL of a 100 M DPPH solution for analysis. At 517 nm, the reaction mixture was read against a reagent blank after 30 min of dark incubation at 25 °C. Ascorbic acid was employed as the benchmark. Calculated as a proportion of DPPH radicals scavenged by *D. dichotoma* extract, antioxidant activity was stated as follows:

(1) $$\%\;\mathrm{RSA}\;=\frac{\mathrm{AC}\;-\;\mathrm{AS}}{\mathrm{AS}}\times 100,$$

RSA = radical scavenging activity; AC = absorption in control; and AS = absorption in sample.

### 4.7. Antimicrobial Activity

Agar well diffusion methods were used to screen *D. dichotoma* extracts of ethyl acetate and *n*-butanol against four bacterial strains (*Staphylococcus aureus, Bacillus subtilis, Escherichia coli, Pseudomonas aeruginosa*, and one fungal strain, *Candida albicans*) [77,78].

#### 4.7.1. Culture of the Bacteria

To cultivate the bacterial and fungal strains, nutrient agar (NA) media were employed. The necessary amount of media was produced, autoclaved, cooled at 40 °C, and then placed on to sterilized Petri plates, where it was left to harden. Before inoculation, the necessary numbers of test organism colonies were cultivated in the appropriate plates and stored in an incubator for 18 to 24 h at 35 °C. These tests were all conducted in a biosafety cabinet aseptically.

#### 4.7.2. Preparation of Inoculum/Suspension

Freshly cultivated bacteria colonies in the required number of colonies (1.5 × 10^8^ cfu·mL^−1^) were aseptically added to glass vials of normal saline. The solution was vortexed to homogenize the suspension, and the results were compared to the 0.5 McFarland standard turbidity advised by the WHO in 1991 for the antibiotic susceptibility test.

#### 4.7.3. Diameter of Inhibitory Zone (DIZ)

Utilizing cotton swabs, inoculum/suspensions were evenly dispersed throughout the dry surface of nutritional agar plates. The infected plates were allowed to stand for a maximum of 15 min to allow absorption of any excess surface moisture. These procedures were repeated three times, with the plate being rotated through an angle of 60 °C between each streaking. Using a sterile cork borer, four wells, each 6 mm in diameter, were drilled into the inoculation plates. Concentrations of 100 mg mL^−1^, 50 mg mL^−1^, 25 mg mL^−1^, and 12.5 mg mL^−1^ were added to the appropriate wells. To ensure accuracy, the experiment was carried out in triplicate. Standard positive controls for antibacterial and antifungal activities were azithromycin and amphotericin, respectively. The incubator was set at 35 ± 1 °C and the infected plates were placed inside. After 18 to 24 h, the plates were checked for the zone of inhibition (ZOI). Each ZOI’s diameter was measured in millimeters using a digital vernier caliper. For the purpose of calculating the extract’s antibacterial potential, the diameter of the zone of inhibition (ZOI) was measured in millimeters [78].

### 4.8. Hypoglycemic Activity

Rats weighing between 120 and 150 g of either sex were employed in the experiment, and they were procured from the PCSIR Peshawar animal house. Animals were kept at room temperature (25 °C). Equal duration (12/12 h) of light and darkness were provided. The standard procedures as proposed by the Animal Ethical Committee at the Department of Pharmacy, University of Malakand (Ref: DREC/Pharm-DM/DD2-2020) were followed. Diabetes was produced in animals by administering alloxan monohydrate (150 mg kg^−1^) intraperitoneally. Each animal’s dose of alloxan was customized based on body weight, and right before injection, it was dissolved in sterile saline. After three days of alloxan administration, rats with plasma glucose levels of more than 200 mg dL^−1^ were included in the experiment. After 72 h, treatment withplant extract was initiated [79].

### 4.9. Animals Groups

Rats were separated into 9 groups (*n* = 6) for this study.

**Group 1:** The rats included in the study as controls received simply distilled water i/p.

**Group 2:** Diabetic control rats received 150 mg kg^−1^ of alloxan i.p.

**Group 3:** Diabetic rats received glibenclamide (5 mg kg^−1^ i.p.)

**Group 4:** Rats with diabetes were administered a 100 mg kg^−1^ ethyl acetate extract.

**Group 5:** Diabetes rats were administered a 200 mg kg^−1^ ethyl acetate extract.

**Group 6:** Rats with diabetes were administered a 300 mg kg^−1^ ethyl acetate extract.

**Group 7:** Rats with diabetes were administered a 100 mg kg^−1^ *n*-butanol extract.

**Group 8:** Rats with diabetes were administered a 200 mg kg^−1^ *n*-butanol extract.

**Group 9:** Rats with diabetes were administered a 300 mg kg^−1^ *n*-butanol extract.

### 4.10. Statistical Analysis

To show significant differences between the means, the one-way analysis of variance (ANOVA) was conducted using the Statistical Software for Social Sciences (SPSS Inc., ver. 13.0, Chicago, IL, USA). Significant differences were indicated as *p* < 0.05, *p* < 0.01, and *p* < 0.001, and the data were given as averages with standard deviation based on three independent assessments.

## 5. Conclusions

Several phytochemicals were identified by the GC-MS study which might be implicated in the antimicrobial, antioxidant, and antidiabetic properties of the extracts. The availability of functionally bioactive chemicals was shown to be strongly influenced by the type of seaweed used as well as the extraction solvent, as shown by in vitro testing and FT-IR analyses. A potential antioxidant activity was observed in the current study. Therefore, it can be concluded that seaweed can be employed as a potential source of organic antioxidant, antimicrobial, and antidiabetic compounds for functional feed or dietary supplements. The identification, separation, and characterization of the active principles responsible for bioactivity utilizing different solvents should be the subject of further research.

## Figures and Tables

**Table 1:** GC-MS analysis of ethyl acetate extract of *D. dichotoma*.

S.No	Compound Name	R-Time	Area	Area %	Molecular Weight	Molecular Formula
1	Undecane	5.995	879421	8.55	156.31	C_11_H_24_
2	Tridecane, 4,8-dimethyl-	8.187	499686	4.86	212.4146	C_15_H_32_
3	2-propenoic acid, 2-ethylhexyl ester	8.863	232461	2.26	288.4	C_19_H_28_O_2_
4	Hexadecane	10.131	50912	0.49	226.41	C₁₆H₃₄
5	Tridecane	10.592	64603	0.63	184.37	C_13_H_28_
6	Benzoic acid, 2-ethylhexyl ester	20.313	134366	1.31	250.3334	C_15_H_22_O
7	Myristic acid	21.377	810080	7.87	228.37	C_14_H_28_O_2_
8	α-Limonene diepoxide	21.514	214806	2.09	168.23584	C_10_H_16_O_2_
9	Ethyl tridecanoate	22.074	83030	0.81	242.3975	C_15_H_30_O
10	1-Octadecyne	23.043	230461	2.24	250.4626	C_18_H_34_
11	2-Undecanone, 6, 10-dimethyl-	23.146	182825	1.78	198.3449	C_13_H_26_O
12	Pentadecanoic acid	23.423	107508	1.04	242.3975	C_15_H_30_O_2_
13	1-Octadecyne	23.551	53831	0.52	250.4626	C_18_H_34_
14	3,7,11,15-Tetramethyl-2-hexadecen-1-ol	23.915	76648	0.74	296.5310	C_20_H_40_O
15	Pentadecanoic acid, 14-methyl-methyl ester	24.768	195344	1.90	270.4507	C_17_H_34_O_2_
16	9-Hexadecenoic acid	25.071	285274	2.77	254.4082	C_16_H_30_O_2_
17	Hexadecenoic acid, *Z*-11-	25.263	1129414	10.98	254.4082	C_16_H_30_O_2_
18	Tetradecanoic acid	25.533	3475165	33.78	228.3709	C_14_H_28_O_2_
19	*E*-11-Hexadecenoic acid, ethyl ester	25.890	83539	0.81	282.4614	C_18_H_34_O_2_
20	Isoaromadendrene epoxide	26.235	471250	4.58	220.3505	C_15_H_24_O
21	Santalol, E-cis,epi-β	26.964	353654	3.44	220.3505	C_15_H_24_O
22	Epianastrephin	27.705	101313	0.98	194.27	C_12_H_18_O_2_
23	6-Octadecenoic acid, methyl ester, (*Z*)-	28.115	130768	1.27	296.4879	C_19_H_36_O_2_
24	β-Elemene	28.552	66749	0.65	204.35	C_15_H_24_
25	Methyl eicosa-5,8,11,14,17-pentaenoate	28.735	375837	3.65	316.4776	C_21_H_32_O_2_

**Table 2:** GC-MS analysis of *n*-butanol extract of *D. dichotoma*.

S.No	Name	R-Time	Area	Area %	Mol. Weight	Mol. Formula
1	Undecane	5.998	863567	14.98	156.31	C_11_H_24_
2	Dodecane	8.1992	494148	8.57	170.33	C_12_H_26_
3	2-propenoic acid, 2-ethylhexyl ester	8.866	238178	4.13	270.36	C_15_H_26_O_4_
4	Lageracetal	9.508	496742	8.62	202.33	C_12_H_26_O_2_
5	Decane	10.599	85112	1.48	142.28	C₁₀H₂₂
6	Tridecane	15.478	378657	6.57	184.37	C_13_H_28_
7	Benzoic acid, 2-ethylhexyl ester	20.317	73951	1.28	250.3334	C_15_H_22_O
8	Tetradecanoic acid	21.341	167535	2.91	228.37	C_14_H_28_O_2_
9	1-Octadecyne	23.042	170327	2.95	250.4626	C_18_H_34_
10	3,7,11,15-tetramethyle-2-hexadecene-1-ol	23.913	57861	1.00	296.5310	C_20_H_40_O
11	Palmitic acid, methyl ester	24.770	165138	2.86	270.45	C_17_H_34_O_2_
12	Hexadecennoic acid, *Z*-11-	25.224	241443	4.19	254.4082	C_16_H_30_O_2_
13	Cetylic acid	25.463	842809	14.62	256.42	CH_3_(CH_2_)_14_COOH
14	Santalol, E-cis,epi-beta	26.951	197198	3.42	220.3505	C_15_H_24_O
15	Aromadendrene oxide-(2)	27.263	248551	4.31	220.35	C_15_H_24_O
16	6-Octadecenoic acid, methyl ester, (Z)-	28.115	78923	1.37	296.4879	C_19_H_36_O
17	Phytol	28.374	246720	4.28	296.53	C_20_H_40_O
18	9-Hexadecenoic acid	29.404	185272	3.21	254.41	C_16_H_30_O_2_
19	Hexadecenoic acid, *Z*-11-	29.633	532477	9.24	254.4082	C_16_H_30_O

**Table 3:** FTIR spectral peak values and functional groups obtained for the leaf extract (in different solvents) of *D. dichotoma*.

Wave Number Ethyl Acetate cm^−1^	Wave Number of *n*-Butanol cm^−1^	Wave Number of Reference cm^−1^	Functional Group	Expected Phytocompound
2922.16	2922.16	2935–2915	Asymmetric stretching of -CH(CH_2_) vibration	Saturated aliphatic comp. Lipids
1714.72–1705.07	1714.72–1705.07	1800–1600	C=O stretches	Carbonyl
1456.26	1456.26	1432–1621	C=C	Aromatic
1375.25	1375.25	1419–1310	O-H bond alcoholic group	Phenol or tertiary alcohol
1238.30	1238.30	1329–1210	C-O stretch, C-N stretch	Acid, amine
1163.08	1163.08	1300–1150	C-N stretch, C-H wag(-CH, k)	Amine, alkyl halides
1020.34	1024.20	1100–1000	PO3	Phosphate ion
981.77	972.12	1000–675	-C-H bending	Alkene
972.12	-	1000–675	-C-H bending	Alkene
736.81	-	1000–675	-C-H bending	Alkene
721.38	721.38	730–500	C-Cl	Halogen compound (chloro-compound)
667.37	667.37–609.51	550–690	C-I	Halogen compound (chloro-compound, iodo-compound), alkyl halide

**Table 4:** Total phenolic (TPC), total flavonoid content (TFC), and antioxidant activity of samples from *D. dichotoma*.

TPC and TFC			Antioxidant Activity		
Compounds	Ethyl Acetate	*n*-Butanol	Conc. µg/mL	IC_50_	
				Ethyl Acetate	*n*-Butanol
TPC(mg GAE/g)	2.56 ± 0.34	2.51 ± 0.67	25	0.68 ± 0.45	0.11 ± 0.65
			50	2.19 ± 0.87	3.19 ± 1.16
TFC(mg QE/g)	2.11 ± 0.89	2.25 ± 0.28	75	5.25 ± 1.02	6.27 ± 1.70
			100	8.31 ± 1.32	9.35 ± 0.32

Values were recorded as mean and standard deviation from 3 replicates, whereas IC_50_ values werecalculated for all the concentrations.

**Table 5:** Hypoglycemic activity of different solvent extracts of *D. dichotoma*.

Treatments	Blood Glucose (mg dL^−1^)			
	1st Day	3rd Day	6 h	24 h
Saline 10 mL	98.00 ± 10.08	99.17 ± 6.68	98.50 ± 7.48	100.00 ± 8.22
Alloxan 150 mg	106.17 ± 11.81	340.50 ± 7.99	339.17 ± 9.28	288.17 ± 8.59
Glibenclamide 5 mg	111.33 ± 7.28	236.83 ± 9.95	224.67 ± 8.69	107.83 ± 7.78
D.D.E 100 mg/kg	99.67 ± 7.61 *	305.33 ± 7.74 *	305.00 ± 7.59 *	294.00 ± 17.78 *
D.D.E 200 mg/kg	107.50 ± 7.48	293.00 ± 5.93 *	291.00 ± 13.54 *	266.50 ± 7.71 *
D.D.E 300 mg/kg	115.67 ± 4.27	287.00 ± 3.63 *	271.33 ± 8.66 *	235.67 ± 7.00 *
D.D.B 100 mg/kg	104.33 ± 8.62	310.17 ± 6.08 *	248.67 ± 5.68 *	245.83 ± 4.75 *
D.D.B 200 mg/kg	99.33 ± 7.15 *	291.00 ± 2.83 *	239.67 ± 5.13 *	233.67 ± 6.80 *
D.D.B 300 mg/kg	89.00 ± 6.51 *	265.17 ± 5.38 *	229.17 ± 5.34	216.67 ± 9.14 *

D.D.E = *D. dichotoma* ethyl acetate extract, D.D.B = *D. dichotomin* butanol extract. * = significant at α > 0.05. Each value represents the mean ± standard deviation of 6 replicates.

## Data Availability

Data related to this paper are available to researchers upon request.

## Supplementary Materials

The following supporting information can be downloaded at: https://www.mdpi.com/article/10.3390/md21050273/s1, Figure S1. GC-MS spectrum of ethyl acetate extract of *D. dichotoma.* Figure S2. GC-MS spectrum of *n*-butanol extract of *D. dichotoma.* Table S1. Antimicrobial activity of *D. dichotoma* extracts

## References

[1] El-Sheekh M.M., Gharieb M.M., El-Sabbagh S.M., Hamza W.T. (2014). Antimicrobial efficacy of some marine macroalgae of Red Sea. Int. J. Microbiol. Immunol. Res..

[2] Araújo R., Vázquez Calderón F., Sánchez López J., Azevedo I.C., Bruhn A., Fluch S., Garcia Tasende M., Ghaderiardakani F., Ilmjärv T., Laurans M. (2021). Current status of the algae production industry in Europe: An emerging sector of the blue bioeconomy. Front. Mar. Sci..

[3] Lomartire S., Marques J.C., Gonçalves A.M. (2021). An overview to the health benefits of seaweeds consumption. Mar. Drugs.

[4] Güven K.C., Coban B., Özdemir O. (2020). Pharmacology of marine macroalgae. Encycl. Mar. Biotechnol..

[5] Sultana P. (2019). Dietary Effects of Seaweed (*Hypnea musciformis*) on Growth Performance and Blood Parameters in Mice. Master’s Thesis.

[6] Akhoundian M., Safaei N. (2022). Ecological status assessment of eastern coastal waters of Qeshm Island (Persian Gulf, Iran) based on macroalgal assemblages. Ecopersia.

[7] Achmad H., Huldani H., Feby Ramadhany Y. (2020). Antimicrobial Activity and Sulfated Polysaccharides Antibiofilms in Marine Algae Against Dental Plaque Bacteria: A Literature Review. Syst. Rev. Pharm..

[8] Umavandhana R., Jayanthi S. (2018). Analysis of Phytochemical compounds and DPPH radical scavenging activity of *Dictyota dichotoma* and *Halimeda macroloba*. Res. J. Pharm. Technol..

[9] Dixit D., Reddy C., Trivedi M., Gadhavi D.K. (2020). Non-targeted metabolomics approach to assess the brown marine macroalga *Dictyota dichotoma* as a functional food using liquid chromatography with mass spectrometry. Sep. Sci. Plus.

[10] Rushdi M.I., Abdel-Rahman I.A., Attia E.Z., Saber H., Saber A.A., Bringmann G., Abdelmohsen U.R. (2022). The biodiversity of the genus *Dictyota*: Phytochemical and pharmacological natural products prospectives. Molecules.

[11] Shameel M., Aisha K., Khan S. (1996). A preliminary survey of seaweeds from the coast of Makran, Pakistan. Bot. Mar..

[12] Manivannan K., Thirumaran G., Karthikai Devi G., Hemalatha A., Anantharaman P. (2008). Biochemical composition of seaweeds from Mandapam coastal regions along Southeast Coast of India. Am.-Eurasian J. Bot..

[13] Abdelrheem D.A., Rahman A.A., Elsayed K.N.M., Ahmed S.A. (2020). GC/MS spectroscopic approach, antimicrobial activity and cytotoxicity of some marine macroalgae from Qusier and Marsa Alam Seashore (RedSea), Egypt. Egypt. J. Aquat. Biol. Fish..

[14] El-Nashar H.A.S., Mostafa N.M., El-Shazly M., Eldahshan O.A. (2021). The Role of Plant-Derived Compounds in Managing Diabetes Mellitus: A Review of Literature from 2014 to 2019. Curr. Med. Chem..

[15] Ślusarczyk J., Adamska E., Czerwik-Marcinkowska J. (2021). Fungi and algae as sources of medicinal and other biologically active compounds: A review. Nutrients.

[16] Hayes M. (2021). Bioactive peptides in preventative healthcare: An overview of bioactivities and suggested methods to assess potential applications. Curr. Pharm. Des..

[17] Manivannan K., Thirumaran G., Devi G.K., Anantharaman P., Balasubramanian T. (2009). Proximate composition of different group of seaweeds from Vedalai Coastal waters (Gulf of Mannar): Southeast Coast of India. Middle-East J. Sci. Res..

[18] Zohra T., Ovais M., Khalil A.T., Qasim M., Ayaz M., Shinwari Z.K., Ahmad S., Zahoor M. (2019). Bio-guided profiling and HPLC-DAD fingerprinting of *Atriplex lasiantha* Boiss. BMC Complement. Altern. Med..

[19] Anjum S., Tahir H., Sarwar S., Raza W., Latif I., Rasheed H.M.F., Jabeen Q., Shahid W., Ashraf M., Zehra S.S. (2022). LC-ESI-MS analysis, antioxidant, anti-diabetic and molecular docking studies on *Corchorus depressus* (L.) C.Chr. Nat. Prod. Res..

[20] Tyagi T., Agarwal M. (2017). Phytochemical screening and GC-MS analysis of bioactive constituents in the ethanolic extract of *Pistia stratiotes* L. and *Eichhornia crassipes* (Mart.) solms. J. Pharmacogn. Phytochem..

[21] Parthiban C., Saranya C., Girija K., Hemalatha A., Suresh M., Anantharaman P. (2013). Biochemical composition of some selected seaweeds from Tuticorin coast. Adv. Appl. Sci. Res..

[22] El-Shenody R.A., Ashour M., Ghobara M.M.E. (2019). Evaluating the chemical composition and antioxidant activity of three Egyptian seaweeds: *Dictyota dichotoma, Turbinaria decurrens,* and *Laurencia obtusa*. Braz. J. Food Technol..

[23] Fraly Erbabley N.Y.G., Junianto J. (2020). Chemical characteristics and phytochemicals of the brown alga *Sargassum filipendulla* from Kelanit waters of southeast Maluku. Egypt. J. Aquat. Biol. Fish..

[24] Mwalugha H.M., Wakibia J.G., Kenji G.M., Mwasaru M.A. (2015). Chemical composition of common seaweeds from the Kenya Coast. J. Food Res..

[25] Gokulakrishnan S., Raja K., Sattanathan G., Subramanian J. (2015). Proximate composition of biopotential seaweeds from Mandapam South East coast of India. Int. Lett. Nat. Sci..

[26] Deyab M.A., El-Katony T.M., El-Adl M.F., Ward F.M. (2017). Temporal variation in chemical composition of *Dictyota dichotoma* (Hudson) JV Lamouroux (Dictyotales, Phaeophyceae) from Red Sea Coast, Egypt. J. Coast. Life Med..

[27] Liu H.-x., Sun S.-q., Lv G.-h., Chan K.K. (2006). Study on Angelica and its different extracts by Fourier transform infrared spectroscopy and two-dimensional correlation IR spectroscopy. Spectrochim. Acta Part A Mol. Biomol. Spectrosc..

[28] Sumathi R., Anuradha R. (2016). FT-IR Spectroscopic Studies on Flowers of *Allamanda neriifolia* Hook. Int. J. Curr. Microbiolgy Appl. Sci..

[29] Potapovich M.V., Kurchenko V.P., Metelitsa D.I., Shadyro O.I. (2011). Antioxidant activity of oxygen-containing aromatic compounds. Prikl. Biokhimiia Mikrobiol..

[30] Abdallah S.H., Mostafa N.M., Mohamed M.A.E.H., Nada A.S., Singab A.N.B. (2022). UPLC-ESI-MS/MS profiling and hepatoprotective activities of *Stevia* leaves extract, butanol fraction and stevioside against radiation-induced toxicity in rats. Nat. Prod. Res..

[31] Mostafa N.M., Edmond M.P., El-Shazly M., Fahmy H.A., Sherif N.H., Singab A.N.B. (2022). Phytoconstituents and renoprotective effect of *Polyalthia longifolia* leaves extract on radiation-induced nephritis in rats via TGF-β/smad pathway. Nat. Prod. Res..

[32] Fischer C.L. (2020). Antimicrobial Activity of Host-Derived Lipids. Antibiotics.

[33] Laus G., Attaur R. (2001). Biological activities of natural halogen compounds. Studies in Natural Products Chemistry.

[34] Farvin K.S., Jacobsen C. (2013). Phenolic compounds and antioxidant activities of selected species of seaweeds from Danish coast. Food Chem..

[35] Imran M., Ullah F., Sadiq A., Ayaz M., Ahmad S., Kamal Z., Zeb A. (2014). Investigation of total phenolic contents, antibacterial, antifungal and anthelmintic potentials of crude methanolic extract, subsequent fractions and crude saponins of *Nonea micrantha* Boiss. & Reut. Pharmacologyonline.

[36] Rehman H., Shah M., Shinwari Z.K., Ali W., Zaman N., Khan M.A., Bibi N.S., Ayaz M. (2022). Total Phenolic-Flavonoid Contents, Anti-Leishmanial, Antimicrobial And Antioxidant Potentials of Pakistani Tea Brands and Tea Plant *Camellia sinensis*. Pak. J. Bot.

[37] Cadar E., Sirbu R., Ibram A., Ionescu A.M. (2019). Evaluation of total phenolic content in relation to antioxidant activity of brown algae *Cystoseira barbata* from Black Sea. Rev. Chim. Buchar. Orig. Ed..

[38] Deyab M., Elkatony T., Ward F. (2016). Qualitative and quantitative analysis of phytochemical studies on brown seaweed, *Dictyota dichotoma*. Int. J. Eng. Dev. Res..

[39] Ozgun S., Turan F. (2015). Biochemical composition of some brown algae from Iskenderun Bay, the northeastern Mediterranean coast of Turkey. J. Black Sea/Mediterr. Environ..

[40] Ktari L., Mdallel C., Aoun B., Chebil Ajjabi L., Sadok S. (2021). Fucoxanthin and phenolic contents of six Dictyotales from the Tunisian Coasts with an emphasis for a green extraction using a supercritical CO_2_ method. Front. Mar. Sci..

[41] Emam M., Mansour H., Shaaban A., Mostafa N. (2014). Biochemical constituents and antioxidant capacity of some seaweeds from red and Mediterranean coasts of Egypt. Egypt. J. Bot..

[42] Kosanić M., Ranković B., Stanojković T. (2019). Brown macroalgae from the Adriatic Sea as a promising source of bioactive nutrients. J. Food Meas. Charact..

[43] Ali S.I., El-Baz F.K., El-Emary G.A., Khan E.A., Mohamed A.A. (2014). HPLC-analysis of polyphenolic compounds and free radical scavenging activity of pomegranate fruit (*Punica granatum* L.). Int. J. Pharm. Clin. Res..

[44] Otero P., Quintana S.E., Reglero G., Fornari T., García-Risco M.R. (2018). Pressurized Liquid Extraction (PLE) as an innovative green technology for the effective enrichment of galician algae extracts with high quality fatty acids and antimicrobial and antioxidant properties. Mar. Drugs.

[45] Benfares R., Kord A., Boudjema K., Bouarab M., Benrabah S., Boudjemaa K., Švarc-Gajić J. (2019). Chemical characterization of essential oils and antioxidant activity of *Dictyota dichotoma* and *Dictyopteris membranacea*. Acta Period. Technol..

[46] Zubia M., Fabre M.S., Kerjean V., LeLann K., Stiger-Pouvreau V., Fauchon M., Deslandes E. (2009). Antioxidant and antitumoural activities of some Phaeophyta from Brittany coasts. Food Chem..

[47] Gammone M.A., Riccioni G., D’Orazio N. (2015). Marine carotenoids against oxidative stress: Effects on human health. Mar. Drugs.

[48] Sachindra N.M., Sato E., Maeda H., Hosokawa M., Niwano Y., Kohno M., Miyashita K. (2007). Radical scavenging and singlet oxygen quenching activity of marine carotenoid fucoxanthin and its metabolites. J. Agric. Food Chem..

[49] Mohamed A.A., Sameeh M.Y., El-Beltagi H.S. (2022). Preparation of Seaweed Nanopowder Particles Using Planetary Ball Milling and Their Effects on Some Secondary Metabolites in Date Palm (*Phoenix dactylifera* L.) Seedlings. Life.

[50] Jebakumar Solomon R.D., Satheeja Santhi V. (2008). Purification of bioactive natural product against human microbial pathogens from marine seaweed *Dictyota acutiloba* J. Ag. World J. Microbiol. Biotechnol..

[51] Demirel Z., Yilmaz-Koz F.F., Karabay-Yavasoglu U.N., Ozdemir G., Sukatar A. (2009). Antimicrobial and antioxidant activity of brown algae from the Aegean Sea. J. Serb. Chem. Soc..

[52] Paz E., Lacy R., Bakhtian M. (1995). The B-Lactum Antibiotics Penicillin and Cephalosporin in Perspective.

[53] Mashjoor S., Yousefzadi M., Esmaeili M.A., Rafiee R. (2016). Cytotoxicity and antimicrobial activity of marine macroalgae (Dictyotaceae and Ulvaceae) from the Persian Gulf. Cytotechnology.

[54] TÜney İ., Cadirci B.H., Ünal D., Sukatar A. (2006). Antimicrobial activities of the extracts of marine algae from the coast of Urla (Izmir, Turkey). Turk. J. Biol..

[55] Durairaj S.B., Andiyappan B.R. (2020). Screening of Phytochemicals, Antibacterial, Antioxidant and Anti-inflammatory Activity of *Dictyota barteyresiana* Seaweed Extracts. Asian J. Biol. Life Sci..

[56] Susanto A., Setyati W.A., Pramesti R., Pringgenies D., Zainuddin M. (2020). Multidrug-Resistant Antibacterial Activity and Active Compound Analysis Several Types of Seaweed from Karimunjawa, Jepara.

[57] Premathilaka R., Silva M. (2016). Bioactive compounds and antioxidant activity of *Bunchosia armenica*. World J. Pharm. Pharm. Sci..

[58] Islam M.S., Al-Majid A.M., Sholkamy E.N., Yousuf S., Ayaz M., Nawaz A., Wadood A., Rehman A.U., Verma V.P., Bari A. (2022). Synthesis, molecular docking and enzyme inhibitory approaches of some new chalcones engrafted pyrazole as potential antialzheimer, antidiabetic and antioxidant agents. J. Mol. Struct..

[59] Zhao C., Yang C., Liu B., Lin L., Sarker S.D., Nahar L., Yu H., Cao H., Xiao J. (2018). Bioactive compounds from marine macroalgae and their hypoglycemic benefits. Trends Food Sci. Technol..

[60] Barzkar N., Jahromi S.T., Poorsaheli H.B., Vianello F. (2019). Metabolites from marine microorganisms, micro, and macroalgae: Immense scope for pharmacology. Mar. Drugs.

[61] Mostafa N.M., Mostafa A.M., Ashour M.L., Elhady S.S. (2021). Neuroprotective effects of black pepper cold-pressed oil on scopolamine-induced oxidative stress and memory impairment in rats. Antioxidants.

[62] Moussa A.Y., Mostafa N.M., Singab A.N.B. (2020). Pulchranin A: First report of isolation from an endophytic fungus and its inhibitory activity on cyclin dependent kinases. Nat. Prod. Res..

[63] Edmond M.P., Mostafa N.M., El-Shazly M., Singab A.N.B. (2021). Two clerodane diterpenes isolated from *Polyalthia longifolia* leaves: Comparative structural features, anti-histaminic and anti-*Helicobacter pylori* activities. Nat. Prod. Res..

[64] Mopuri R., Ganjayi M., Meriga B., Koorbanally N.A., Islam M.S. (2018). The effects of *Ficus carica* on the activity of enzymes related to metabolic syndrome. J. Food Drug Anal..

[65] Ayoub N., Singab A.N., Mostafa N., Schultze W. (2010). Volatile constituents of leaves of *Ficus carica* Linn. Grown in Egypt. J. Essent. Oil-Bear. Plants.

[66] Ahmad I., Ahmed S., Akkol E.K., Rao H., Shahzad M.N., Shaukat U., Basit A., Fatima M. (2022). GC–MS profiling, phytochemical and biological investigation of aerial parts of *Leucophyllum frutescens* (Berl.) IMJohnst (Cenizo). S. Afr. J. Bot..

[67] Saleem U., Akhtar R., Anwar F., Shah M.A., Chaudary Z., Ayaz M., Ahmad B. (2021). Neuroprotective potential of *Malva neglecta* is mediated via down-regulation of cholinesterase and modulation of oxidative stress markers. Metab. Brain Dis..

[68] Ahmad S., Ullah F., Ayaz M., Ahmad A., Sadiq A., Mohani S.N.-U.-H. (2019). Nutritional and medicinal aspects of *Rumex hastatus* D.Don along with in vitro anti-diabetic activity. Int. J. Food Prop..

[69] Onwuka G.I. (2005). Food Analysis and Instrumentation: Theory and Practice.

[70] Mostafa N.M. (2018). β-Amyrin Rich *Bombax ceiba* Leaf Extract with Potential Neuroprotective Activity against Scopolamine-Induced Memory Impairment in Rats. Rec. Nat. Prod..

[71] Younis M.M., Ayoub I.M., Mostafa N.M., Al-Rashood S.T., Eldahshan O.A. (2022). GC/MS Profiling, anti-collagenase, anti-elastase, anti-hyaluronidase activities of a *Stenocarpus sinuatus* leaves extract. Plants.

[72] Ayaz M., Junaid M., Ullah F., Sadiq A., Shahid M., Ahmad W., Ullah I., Ahmad A., Syed N.-i.-H. (2017). GC-MS analysis and gastroprotective evaluations of crude extracts, isolated saponins, and essential oil from *Polygonum hydropiper* L. Front. Chem..

[73] Zohra T., Ovais M., Khalil A.T., Qasim M., Ayaz M., Shinwari Z.K. (2019). Extraction optimization, total phenolic, flavonoid contents, HPLC-DAD analysis and diverse pharmacological evaluations of *Dysphania ambrosioides* (L.) Mosyakin & Clemants. Nat. Prod. Res..

[74] Odabasoglu F., Aslan A., Cakir A., Suleyman H., Karagoz Y., Halici M., Bayir Y. (2004). Comparison of antioxidant activity and phenolic content of three lichen species. Phytother. Res..

[75] Shah S.M., Ayaz M., Khan A.-U., Ullah F., Farhan, Shah A.-U.-H.A., Iqbal H., Hussain S. (2015). 1,1-Diphenyl,2-picrylhydrazyl free radical scavenging, bactericidal, fungicidal and leishmanicidal properties of *Teucrium stocksianum*. Toxicol. Ind. Health.

[76] Shah S.M., Ullah F., Ayaz M., Wahab A., Shinwari Z.K. (2019). Phytochemical profiling and pharmacological evaluation of *Ifloga spicata* (forssk.) Sch. Bip. in leishmaniasis, lungs cancer and oxidative stress. Pak. J. Bot..

[77] Sani A., Hassan D., Khalil A.T., Mughal A., El-Mallul A., Ayaz M., Yessimbekov Z., Shinwari Z.K., Maaza M. (2021). Floral extracts-mediated green synthesis of NiO nanoparticles and their diverse pharmacological evaluations. J. Biomol. Struct. Dyn..

[78] Ayaz M., Subhan F., Ahmed J., Khan A.-U., Ullah F., Sadiq A., Syed N., Ullah I., Hussain S. (2015). Citalopram and venlafaxine differentially augments antimicrobial properties of antibiotics. Acta Pol. Pharm. Drug Res..

[79] Sadiq A., Rashid U., Ahmad S., Zahoor M., AlAjmi M.F., Ullah R., Noman O.M., Ullah F., Ayaz M., Khan I. (2020). Treating hyperglycemia from *Eryngium caeruleum* M.Bieb: In-vitro α-glucosidase, antioxidant, in-vivo antidiabetic and molecular docking-based approaches. Front. Chem..

