# Supplementary material for: Chemical and Nutritional Profiling of the Seaweed Dictyota dichotoma and Evaluation of Its Antioxidant, Antimicrobial and Hypoglycemic Potentials
Source: Mar Drugs. 2023 Apr 27;21(5):273. doi: 10.3390/md21050273 (PMC10223360; doi:10.3390/md21050273)
Supplement: Supplementary file 1 [file marinedrugs-21-00273-s001.zip › marinedrugs-2321448-supplementary.pdf]

marine drugs

MDPI

Supplementary File

Article

Chemical and Nutritional Profiling of the Seaweed *Dictyota dichotoma* and Evaluation of Its Antioxidant, Antimicrobial and Hypoglycemic Potentials

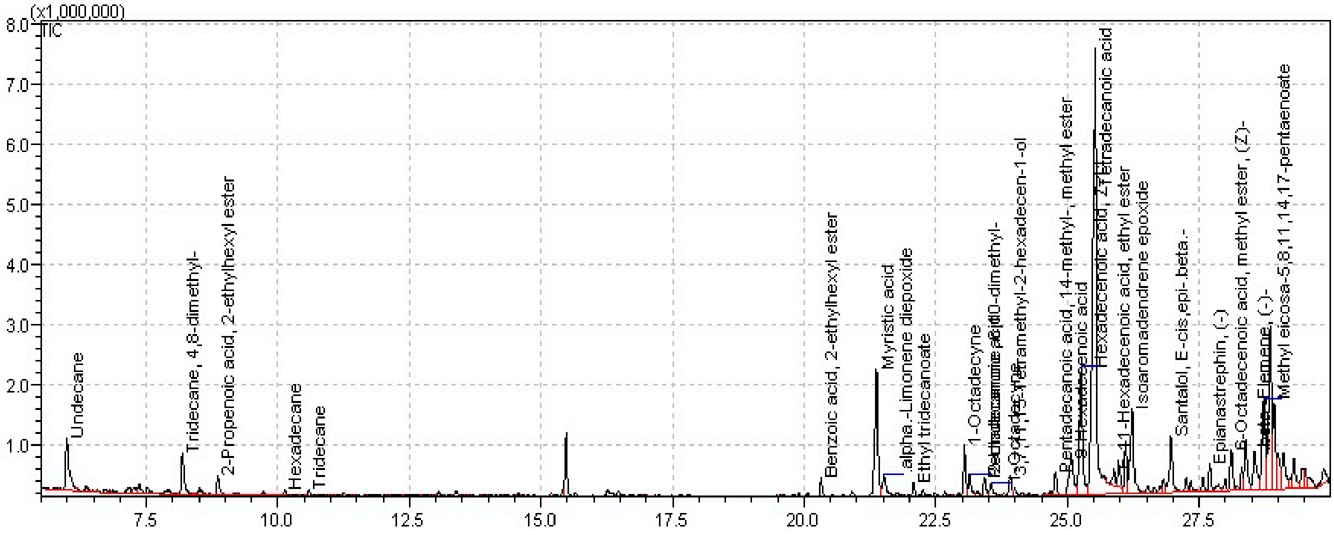

Figure S1. GC-MS spectrum of ethyl acetate extract of *D. dichotoma*.

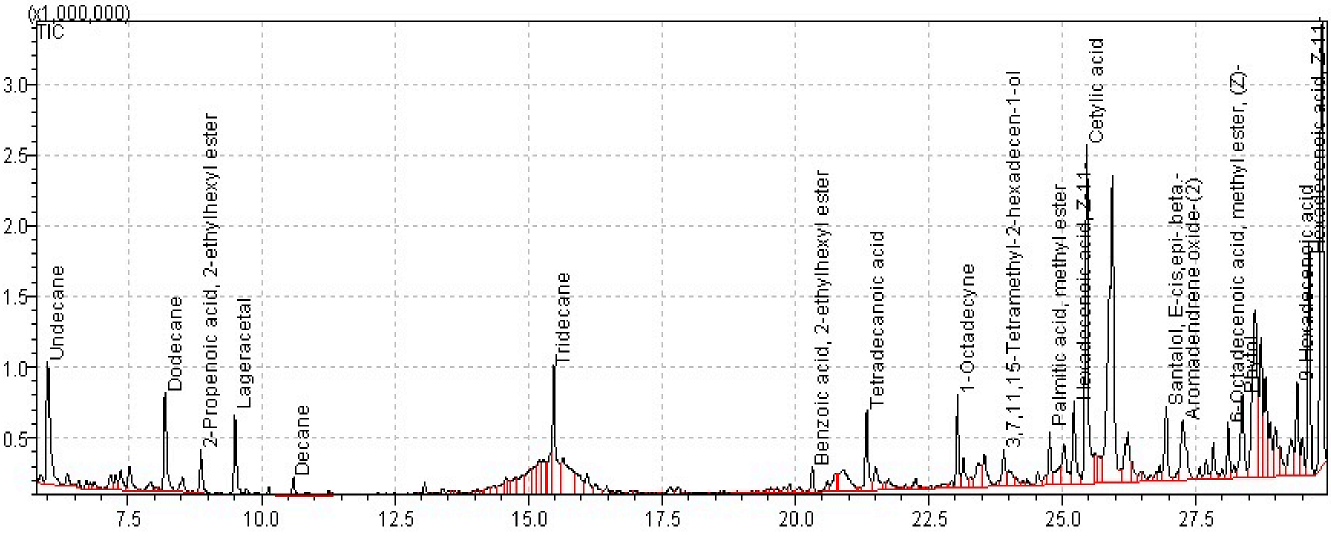

Figure S2. GC-MS spectrum of *n*-butanol extract of *D. dichotoma*.

marine drugs

MDPI

Table S1. Antimicrobial activity of *D. dichotoma* extracts.

Extracts	Dose mg/ml	Inhibition zone diameter (mm)				
		E. coli	*P. aeruginosa*	*S. aureus*	*B. subtilis*	*C. albicans*
Azithromycin, amphotericin	Control	36.33±0.58	38.00±1.00	32.67±1.53	27.67±1.53	23.00±1.00
Ethyl acetate	100mg/ml	16.33±1.53[c]	13.33±0.58[c]	15.33±0.58[c]	14.00±1.00[c]	12.67±2.08[a]
	50mg/ml	15.00±1.00[c]	9.00±7.81[c]	14.00±1.00[c]	12.67±0.58[c]	11.67±0.58[a]
	25mg/ml	14.00±1.00[c]	8.33±7.23[c]	13.67±1.53[c]	12.67±0.58[c]	10.67±0.58[a]
	12.5mg/ml	14.00±1.00[c]	7.67±6.66[c]	13.33±1.53[c]	8.33±7.23[c]	7.00±6.08[a]
***n*-butanol**	100mg/ml	16.00±1.73[c]	15.00±0.00[c]	12.00±0.00[c]	15.33±1.53[c]	17.33±2.08[b]
	50mg/ml	15.67±1.15[c]	13.33±0.58[c]	10.67±0.58[c]	13.67±1.15[c]	16.33±2.52[b]
	25mg/ml	14.67±1.53[c]	9.00±7.81[c]	07.33±6.35[c]	13.33±0.58[c]	15.33±2.52[c]
	12.5mg/ml	13.67±0.58[c]	0.00±0.00[c]	6.33±5.51[c]	12.33±0.58[c]	15.00±3.00[c]

a) significantly different at $\alpha < 0.001$, 0.01 and 0.05; **b)** significantly different at $\alpha < 0.01$; **c)** significantly different at $\alpha < 0.05$. Each value was recorded as mean and standard deviation from 3 replicates.
